# Mitochondrial dysfunction in peripheral mononuclear blood cells (PBMC) of individuals with mild cognitive impairment

**DOI:** 10.1007/s11357-025-01813-4

**Published:** 2025-08-06

**Authors:** Fabian Dieter, Karlotta Jacobs, Alice Quentin, David Prvulovic, Andreas Reif, Johannes Pantel, Ulrich Pilatus, Elke Hattingen, Silke Matura, Gunter Peter Eckert

**Affiliations:** 1 Institute of Nutritional Science, Justus-Liebig University, Giessen, Germany; 2 Department of Psychiatry, Psychosomatic Medicine and Psychotherapy, Goethe University Frankfurt, University Hospital, Frankfurt am Main, Germany; 3 Geriatric Medicine, Institute of General Practice, Goethe University, Frankfurt am Main, Germany; 4 Institute for Neuroradiology, University Hospital, Goethe University, Frankfurt am Main, Germany; 5 Brain Imaging Center (BIC), University Hospital Frankfurt, Frankfurt am Main, Germany

**Keywords:** Mild cognitive impairment, Mitochondrial function, Aging, PBMC

## Abstract

Mitochondrial dysfunction is a hallmark of aging and many age-related neurodegenerative diseases. Mild cognitive impairment (MCI) refers to a clinical condition characterized by noticeable cognitive decline that exceeds normal age-related changes but does not significantly interfere with daily functioning. MCI is often considered an early stage of neurodegenerative conditions, including Alzheimer’s disease. We therefore investigated the relationship between mitochondrial function in peripheral blood cells and cognitive performance in individuals with amnestic (aMCI) and nonamnestic mild cognitive impairment (naMCI). Control groups consisted of young (YC) and older adults (OC) who were physically and mentally healthy. Cross-sectional observational study involving 90 participants, including young adults, cognitively healthy older adults, and individuals with MCI. Mitochondrial function was determined in cryopreserved PBMCs. Cognitive status was assessed using the German version of the Consortium to Establish a Registry for Alzheimer’s Disease (CERAD) test battery. ATP levels in cryopreserved PBMC isolated from individuals with aMCI were significantly lower than those of OC and YC. Endogenous respiration varied significantly between groups, with the MCI group exhibiting the lowest respiration. Linear regression analyses with ATP as a predictor for cognitive performance showed a significant positive relationship between ATP levels and both immediate recall and fluency. The regression coefficients indicated a moderate positive correlation between ATP levels and performance in both tests. This suggests that higher ATP levels are associated with improved cognitive performance. Our data suggest that mitochondrial dysfunction in PBMC is associated with MCI and correlates with cognitive impairment. Subjects who performed poorly on neuropsychological tests also exhibited lower ATP levels. Given that PBMC are easily accessible, they offer valuable insights into the bioenergetic status of individuals at increased risk for dementia. The study (PEM-MCI) has been retrospectively registered at the German Register of Clinical Trials (DRKS) DRKS00036017 (registered on 30.01.2025).

**Graphical Abstract:**

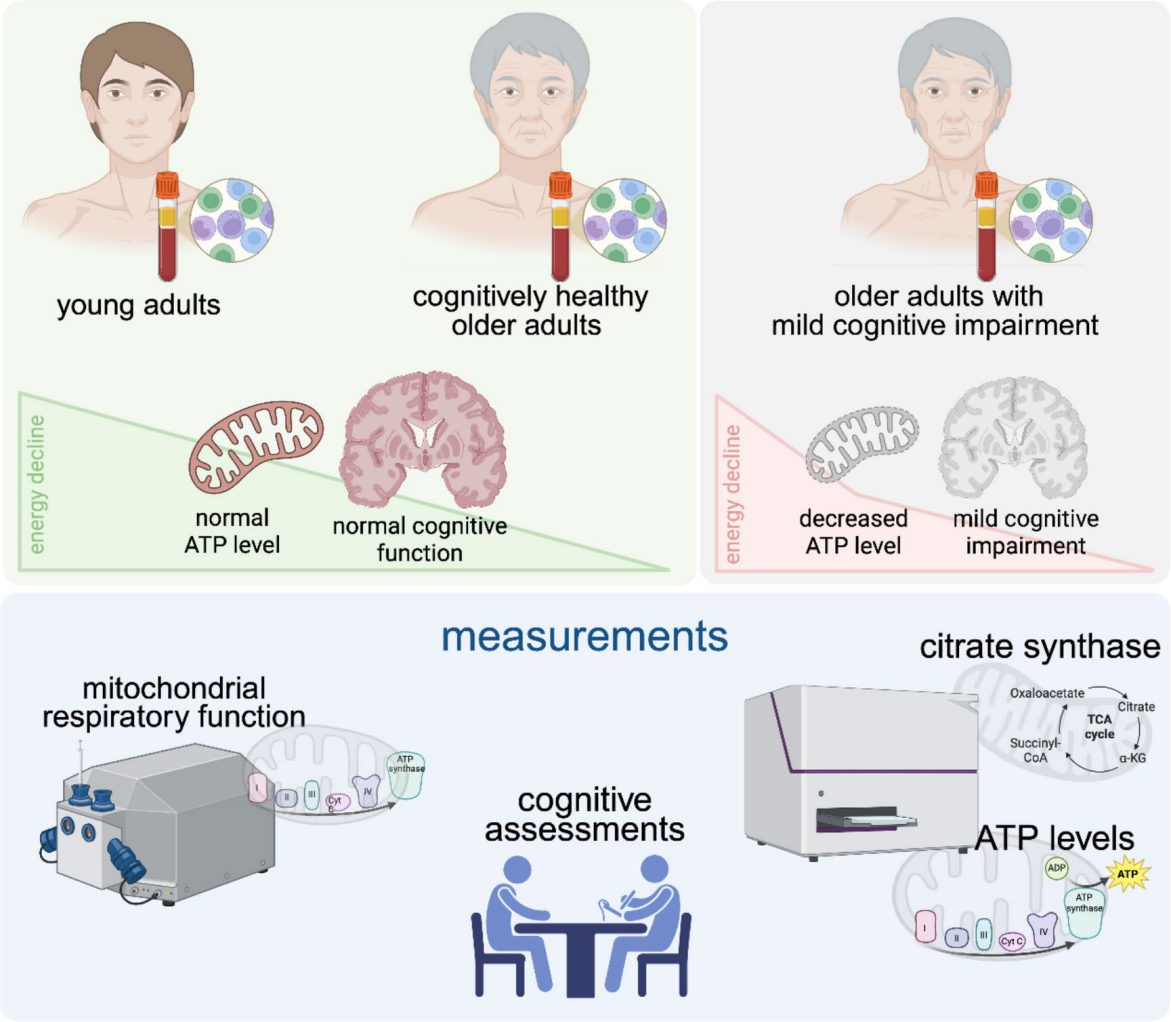

**Supplementary Information:**

The online version contains supplementary material available at 10.1007/s11357-025-01813-4.

## Introduction

Mitochondria play a crucial role in aging [1]. Thus, mitochondrial function is essential for healthy aging, especially in tissues having high energy demands. Although the human brain weighs only 1.2 to 1.5 kg, it consumes at least 20% of the body’s total energy [2]. Cerebral mitochondria provide energy essential for brain function, playing a critical role in regulating synaptic transmission and supporting cognitive processes [3, 4]. Factors such as mitochondrial DNA replication [5, 6], the generation of reactive oxygen species (ROS) [7] and impaired oxidative phosphorylation [8–10] contribute significantly to age-related mitochondrial dysfunction, ultimately leading to reduced ATP production [9]. Mitochondrial dysfunction (MD) plays an important role in age-related diseases, particularly in post-mitotic tissues, including the brain, and is strongly associated with neurodegeneration [11, 12]. As a result of aging and the associated MD, individuals experience a decline in cognitive function, showing a similar pattern of metabolic changes in mild cognitive impairment (MCI) and Alzheimer’s disease (AD) in in vivo magnetic resonance spectroscopy studies [13]. Based on preclinical data, MD has been identified as a common final pathway in brain aging and Alzheimer’s disease [14, 15]. A recent study reported that brain energy metabolism and mitochondrial function were greater in temporal than bifrontal regions in brains of older adults with memory complaints and a first-degree family history of AD using 31P MRS measurements. However, findings were not related to the cognition in older adults [16]. Using H1- and 31P- MRS measurements, we have recently demonstrated that N-acetylaspartate and N-acetylaspartate-glutamate (tNAA) levels were reduced, creatine (Cr) and phosphor-creatine (PCr) levels were increased, and ATP levels were unchanged in brains of cognitively healthy older adults [17]. In this study, we also have shown that ATP levels and respiratory chain function are reduced in peripheral blood mononuclear cells (PBMC) isolated from cognitively healthy elderly compared to a young control group [17].

Based on previous findings [18] and on the basis of our results, we extended our studies to investigate whether the age-related decline in peripheral mitochondrial function in people suffering from mild cognitive impairment is accompanied by further deterioration.

For this purpose, PBMC were obtained from individuals with amnestic MCI (aMCI) and nonamnestic mild cognitive impairment (naMCI) and their mitochondrial function was compared with that of PBMC from age-matched healthy controls and healthy young subjects. Cognitive performance was assessed using a neuropsychological test battery. We hypothesized that individuals with mild cognitive impairment exhibit impaired mitochondrial function, which is likely associated with their cognitive performance.

## Methods

### Study design and participants

**Fig. 1:**
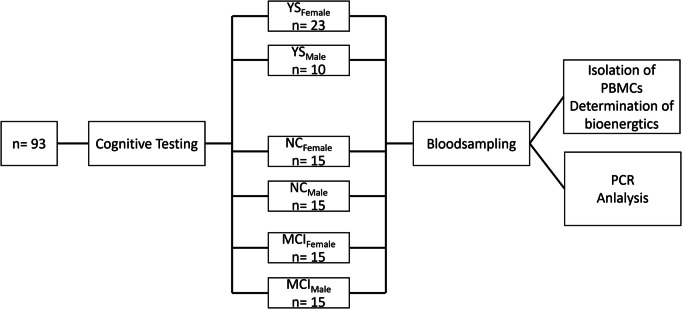
Study design. A total of 93 subjects were recruited to participate in this study. No participant had to be excluded; none withdrew from the study

The study included ninety male and female volunteers aged between 20 and 83 years. The two groups of older participants were age- and gender-matched, with 15 female and 15 male participants per group. The younger subjects were composed of 7 male and 23 female participants. The study design is illustrated in Fig. 1. The Ethics Committee of the Goethe University Frankfurt, Germany, approved the study design (reference 2021–277), which was performed in agreement with the Declaration of Helsinki (Version Fortaleza 2012). Study participants were informed about the upcoming psychological assessments and blood collection. Individuals with MCI and the control group were matched for age and gender. They were recruited through the memory clinic of the Department of Psychiatry, Psychosomatics and Psychotherapy at Goethe University Hospital in Frankfurt am Main. The control group of young participants consisted of students from Justus Liebig University in Giessen who did not undergo neurocognitive testing. Written informed consent was obtained from all participants after they were given information about the course of the study. The blood samples were drawn at 8 a.m., after a twelve-hour fasting period. Exclusion criteria included neurological diseases, diagnosis of lifetime bipolar I disorder, schizophrenia, organic mental disorders, or substance abuse hemophilia, haematophobia, abnormalities in hematology, or intake of anticoagulants. The study (PEM-MCI) has been retrospectively registered at the German Register of Clinical Trials (DRKS) DRKS00036017 (registered on 30.01.2025).

### Neuropsychological tests

Various cognitive tests from the Consortium to Establish a Registry for Alzheimer ‘s Disease (CERAD; German Version) were used to assess cognitive performance. The CERAD test, which comprises a total of 11 subtests, includes five tests relevant to this study: the Animal Fluency Test (AFT) and Semantic Fluency Test (SFT) measure executive function and linguistic performance [19]. They were combined into one category “Fluency”. The Immediate Recall (IR) and Delayed Recall (DR) both measure episodic memory for different time intervals [20]. Finally, the Trail Making Test—Part B (TMT-B) was included to measure executive functions, specifically cognitive flexibility and attention [21].

### Peripheral blood mononuclear cell isolation

PBMC were isolated from venous blood as described [22]. Briefly, blood was taken from a suitable vein and collected in a lithium-heparin monovette (Sarstedt). The PBMC were then isolated from the blood using density centrifugation. Biocoll separating solution with a density of 1.077 g/ml (Bio&Sell. Biocoll®, 1.077 g/ml) was used for the isolation [23]. For isolation, the blood was mixed 1:1 with Dulbecco’s phosphate-buffered saline solution (DPBS) (without calcium and magnesium) and layered on the separating solution, then at room temperature centrifuged at 1000 g for 10 min with the brakes turned off. After separation of the blood components, the PBMC were removed from their phase and transferred to a new 50 ml Falcon. To remove residues of platelets and separating solution from the PBMC, the cell suspension was filled up to 25 ml with DPBS and centrifuged for 10 min at 100 g. The supernatant was removed and the washing step was repeated with 25 ml DPBS [22]. For cryopreservation, the PBMC pellet was resuspended in fetal bovine serum (FBS).

### Cryopreservation

Cryopreservation was performed as recently described [23]. The isolated PBMC were resuspended in 1 ml FBS and transferred to a cryovial. To avoid damage from the freezing process, 1 ml of a mixture of FBS and dimethyl sulfoxide (DMSO) was added dropwise (final DMSO concentration 10%). The vials were transferred to a freezing container and stored at − 80 °C for 24 h. For long-term storage, the PBMC were placed in the gas phase of liquid nitrogen.

### Thawing

A recently published protocol was used to thaw the cells [23]. For thawing, the PBMC were placed in a 37 °C water bath for approx. 3 min. Pre-warmed RPMI medium supplemented with 10% FBS, 50 U/ml penicillin and 50 U/ml streptomycin was added to the cells to dilute the DMSO. To wash the cells, they were centrifuged for 10 min at 100 g, and the supernatant was removed. After the washing step, the cells were resuspended in fresh cell culture medium, counted, and prepared for the corresponding experiments. To allow the cells to recover after cryopreservation, they were stored for 24 h at 37 °C and 5% CO2 saturation in a cell incubator.

### Cell count and viability

To ensure that the PBMC showed high viability, the cell number and viability were determined using the TC20™-cell counter. A living dead discrimination was performed by trypan blue.

### Determination of ATP levels

To determine the total ATP content of the cells, they were seeded in 96-well plates at a density of 100,000 cells per ml. The ATPlite luminescence assay system (6016949, Perkin Elmer, Rodgau-Jügesheim, Germany) was used to determine ATP concentrations using a ClarioStar plate reader (BMG Labtech, Ortenberg, Germany). At the beginning of the measurement, the cells were taken out of the incubator so that they could cool down to room temperature for 15 min. After the plates had cooled down, the cells were lysed for 5 min using lysis solution. By adding the monitoring reagent, the ATP content could be determined by luminescence after 40 min using a calibration curve.

### High resolution respirometry in permeabilized PBMC

**Fig. 2:**
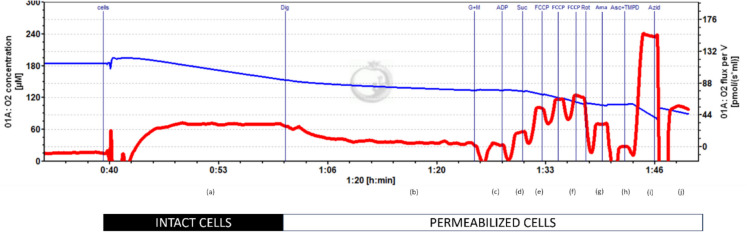
Exemplary measurement curve of respiration. (**a**) Endogenous respiration, (**b**) respiration after permeabilization, (**c**) uncoupled complex I respiration, (**d**) coupled complex I respiration; (**e**) OXPHOS; (**f**) maximal uncoupled activity of CI and CII; (**g**) uncoupled respiration of complex II; (**h**) residual respiration (ROX); (**i**) CIV_(E)_ uncoupled respiration and autooxidation of TMPD; (**j**) autooxidation of TMPD

The activity of the individual complexes of the respiratory chain and their coupled interaction were determined using the Oxygraph-2 k respirometer (Oroboros Instruments, Innsbruck, Austria) and the associated software DatLab v. 7.4.0.4. The protocol used was developed by Erich Gnaiger [24] and is used for live measurement of oxygen consumption in the chambers of the Oxygraph-2 k respirometer. PBMC were resuspended in mitochondrial respiration medium (MIR05) [25]. In the chambers, 2 × 10^6^ cells were added, with a cell density of 4 × 10^6^ cells/ml. Each sample was measured as a duplicate, in parallel in both chambers of the Oxygraph-2 k respirometer. The mean value from both chambers was then calculated and displayed as a result. To see differences between the chambers, the coefficient of variance was determined. The coefficient of variance between the chambers was 7.22 for endogenous respiration, 9.82 for CI(_L_), 9.22 for CI(_P_), 10.3 for CI&CII(_P_), 8.37 for CI&CII(_E_), 10.4 for CII(_E_), and 7.93 for CIV (_E_). To measure the activity of complexes I to IV of the mitochondrial respiratory chain, specific substrates, inhibitors, and uncouplers were used. First, the cells were transferred to the chambers of the electrode to determine their endogenous respiration. In the next step, the cell membrane was permeabilized by digitonin [5 µg/ml] to make the membrane permeable to the substrates and inhibitors. The substrates glutamate 10 mM and malate 4 mM were added to equalize the proton leak across the membrane, and LEAK respiration was measured (CI_(L)_). The addition of ADP [5 mM] measured the coupled respiration of complex I (CI_(P)_). The additional addition of succinate [10 mM] measured the OXPHOS. To measure the maximum activity of the electron transfer system (ETS) FCCP [0,25 µM] was added to saturation. The subsequent addition of rotenone [0,5 µM] was used to differentiate between complex I and complex II activity. Residual oxygen consumption (ROX) was determined by adding antimycin A [0,25 µM]. ROX is non-mitochondrial respiration. Finally, tetramethylphenylenediamine (TMPD) [1 mM] and ascorbate [2,5 mM] were added to measure complex IV activity. To inhibit the respiratory chain, sodium azide [> 100 mM] was added to the chambers; the remaining signal was subtracted from the complex IV activity as it resulted from the autooxidation of TMPD. A sample measurement curve of the Oxygraph-2 k respirometer is illustrated in Fig. 2.

### Citrate synthase activity

Citrate synthase activity was determined using the same method as described here [26]. Briefly summarized, the conversion of DTNB to TNB is used; this conversion was measured at 412 nm in a ClarioStar plate reader (BMG Labtech, Ortenberg, Germany).

### Apolipoprotein E genotype

The ApoE genotype, an important risk factor for Alzheimer’s disease, was determined in PBMC by RT-PCR, particularly as APOE4 homozygotes have recently been identified as a genetic form of Alzheimer’s disease [27]. Total RNA was isolated using the RNeasy Mini Kit (Qiagen, Hilden, Germany) according to the manufacturer’s instructions using ~ 20 mg RNAlater stabilized samples (Qiagen, Hilden, Germany). RNA was quantified by measuring the absorbance at 260 and 280 nm using a NanoDrop™ 2000c spectrometer (Thermo Fisher Scientific, Waltham, MA, USA). RNA purity was assessed using the ratio of absorbance 260/280 and 260/230. To remove residual genomic DNA, samples were treated with a TURBO DNA-free™ kit according to the manufacturer’s instructions (Thermo Fisher Scientific, Waltham, MA, USA). Complementary DNA was synthesized from 250 ng total RNA using the iScript cDNA Synthesis Kit (BioRad, Munich, Germany) according to the manufacturer’s instructions and was stored at − 80 °C. qRT-PCR was conducted using a CfX 96 Connect™ system (BioRad, Munich Germany). APOE genotyping was performed as described by Calero and colleagues [28]. The characteristics of the primers are presented in Supplement-Table 1 for reference.

**Table 1:** Demographic/biological data and neuropsychological test scores. *IR* Immediate Recall, *DR* Delayed Recall, *TMT-B* Trail Making Test B, F*luency* Animal Fluency and Semantic Fluency combined, *aMCI* amnestic mild cognitive impairment, *naMCI* nonamnestic mild cognitive impairment. The values denote the mean raw scores (± standard deviation) or the number of subjects. *N* = sample size. The ***p < 0.0001 is significantly different compared between MCI and age and gender matched Control Group (unpaired *t*-test)

	Young control group	Old control group	MCI-group
	*n* = 33	*n* = 30	*n* = 30 (aMCI = 25, naMCI = 5)
Demographic and biological data			
Age (M/SD)	25.18 ± 3.57	68.90 ± 6.95	71.10 ± 7.92
Gender (female/male)	23/10	15/15	15/15
Education years (M/SD)	18.18 ± 3.57	17.20 ± 2.60	14.80 ± 3.28
Body mass index (M/SD)	23.87 ± 2.11	23.71 ± 4.87	25.34 ± 3.16
APOE genotype		2/3: 5, 2/4: 0, 3/3: 15 3/4: 9, 4/4: 1	2/3: 5, 2/4: 2, 3/3: 15 3/4: 4, 4/4: 3
Neuropsychological tests			
IR (M/SD)		23.66 ± 3.97	17.53 ± 5.11***
DR (M/SD)		8.06 ± 1.38	5.73 ± 2.31***
TMT-B (M/SD)		75.50 ± 24.04	134.46 ± 72.91***
Fluency (M/SD)		40.80 ± 8.69	28.76 ± 11.51***

### Chemicals

The chemicals used for this research were purchased from either Merck, Sigma-Aldrich, Thermo Fisher Scientific, or VWR in the highest purity available.

### Statistics

Unless stated otherwise, data is presented as mean ± SD. Outliers were removed via ROUT (Q = 1%) outlier test. Statistical testing was performed using either Student’s *t*-test, *Mann–Whitney U-test* or a one-way ANOVA followed by a Tukey post hoc test. To analyze the association between cognitive performance and mitochondrial function, separate linear regression analyses were calculated. Age, gender and education were used as covariates for the calculation of linear regressions. The program Prism 8.0 GraphPad Software was used for statistical evaluation. Statistical significance was defined for *p* values: *p < 0.05, **p < 0.01, ****p < 0.0001. The linear regressions were calculated using statistical software Statistica 13.3 V5.

## Results

### Demographic data and neuropsychological test performance

Demographic data of all participants in the 3 different groups are shown in Table 1. Body Mass Index and APOE genotype data are included, along with the neuropsychological data for all elderly participants. The young control group consisted of students who were not neurocognitively tested. The total sample had a mean age of 70 years, with the MCI group being on average 2.2 years older than the healthy participants (MCI: *M* = 71.10, *SD* = 7.92; healthy controls: *M* = 68.90, *SD* = 6.95); however, this difference was not statistically significant. In the MCI group, 25 people were diagnosed with amnestic mild cognitive impairment and 5 people with non-amnestic mild cognitive impairment. Both groups were evenly balanced in terms of gender (15 female/15 male), with the healthy group having a higher average level of education (see Table 1). In terms of neuropsychological performance, the MCI group showed lower scores across all tasks. Marked differences were observed particularly in memory (Immediate and Delayed Recall), executive functions (TMT-B), and verbal fluency (see Table 1). These findings reflect typical cognitive deficits associated with MCI. In the presentation of the results, no distinction was made between aMCI and naMCI; instead, all results were summarized under the term MCI.

### Mitochondrial function in cryopreserved PBMC

#### Effects of cryopreservation on mitochondrial function in PBMC

In the previous study, on which this work is based, the PBMC obtained were freshly measured [17]. In the current study, the PBMC had to be frozen for logistical reasons. However, samples could be affected by the cryopreservation process, by transportation, by changing temperatures during storage, and by the storage temperature itself [29–31]. We therefore verified the procedures before the actual study and isolated and cryopreserved PBMC from young donors using a recently published method [23]. Thus, storing cryopreserved PBMC was therefore unlikely to have affected the results.

#### ATP-level and citrate synthase activity in cryopreserved PBMC

**Fig. 3:**
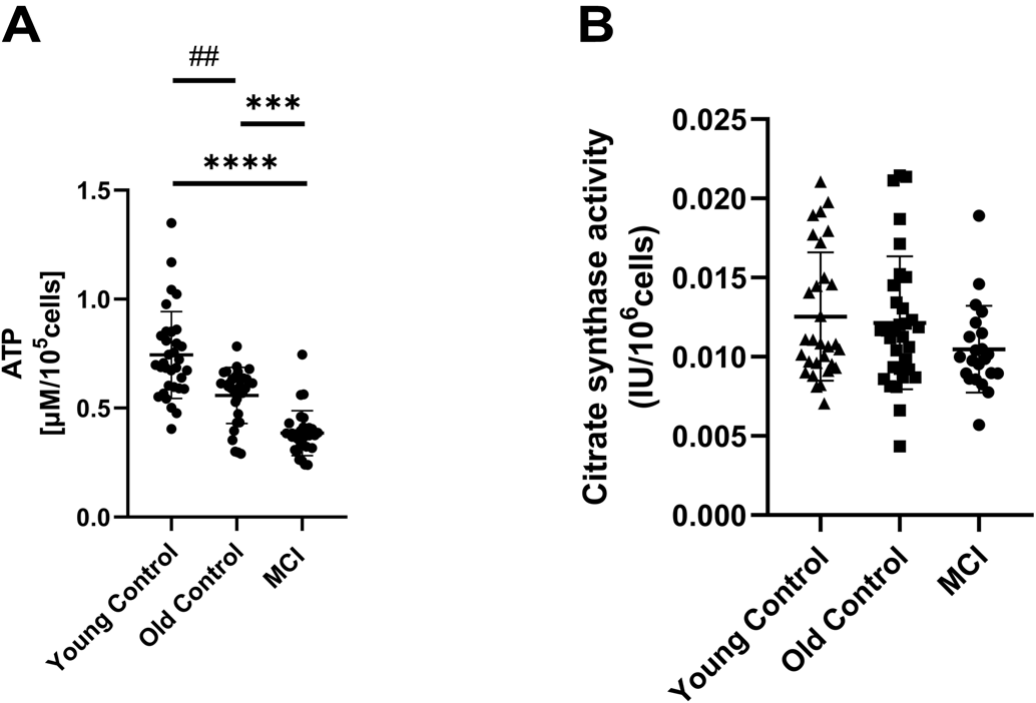
ATP-Level and activity of citrate synthase: **A** ATP values and **B** Citrate synthase activity were measured in cryopreserved PBMC, in young control, older controls, and people with mild cognitive impairment (MCI). *n* = 30 in each group, except ATP level young control = 33. The data are given as mean values ± SD. Statistical significance was tested using an analysis of variance (Friedman test (**A**) and Kruskal–Wallis test (**B**)) (**p < 0.01, ***p < 0.001, ****p < 0.0001)

ATP levels are significantly different in all groups: The mean ATP levels of subjects with MCI were significantly lower than that of controls of the same age (OC) (*p* < 0.0006) and that of young subjects (YC) (*p* < 0.0001). A significant difference was also found between ATP levels in PBMC of healthy young and older subjects (*p* < 0.0025) (Fig. 3A). The activity of citrate synthase did not differ in any of the groups (Fig. 3B). The citrate synthase activity (CS) is used as a marker for mitochondrial mass, which is considered common practice [26].

#### Activity of respiration chain complexes (I-IV) in cryopreserved PBMC

**Fig. 4:**
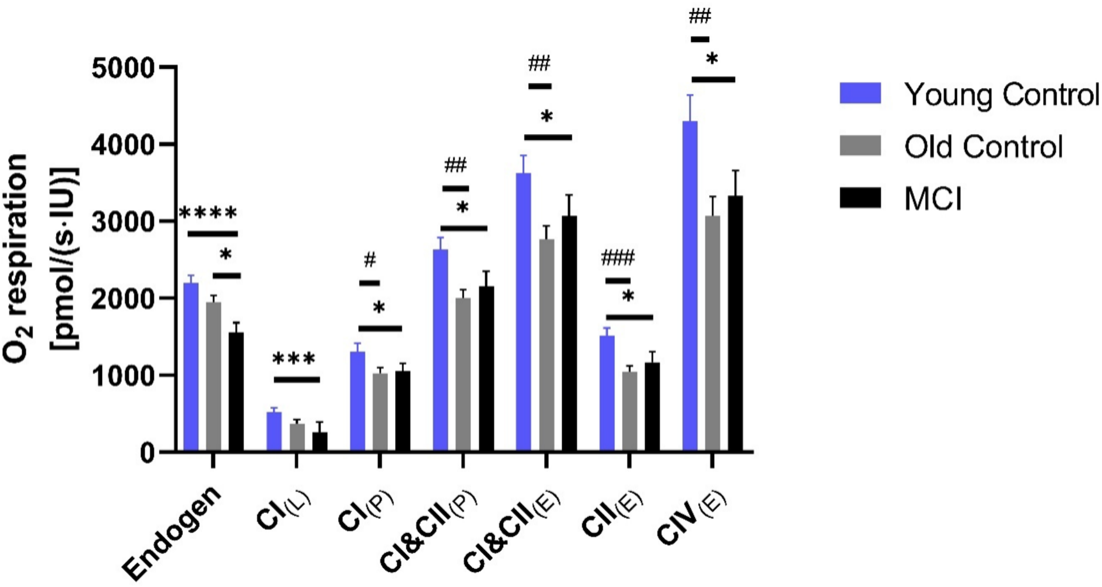
Mitochondrial respiration PBMC were added to the chambers of the Oxygraph-2 k respirometer [4 × 10^6 ^cells/ml] to measure the mitochondrial respiratory chain and its complexes. The activity of citrate synthase was used to normalize the respiration data. Various inhibitors, substrates, and uncouplers were added to differentiate between the individual respiratory chain complexes. Mitochondrial respiration was determined in young control, older controls, and people with mild cognitive impairment (MCI). OC = 30, Yc = 30, MCI = 24. The data are given as mean values ± SEM. Significances were determined with an ANOVA (Kruskal–Wallis test or Friedman test). (MCI vs. the control groups: *p < 0.05, **p < 0.01, ***p < 0.001, ****p < 0.0001; Young vs. old control group: ^#^*p* < 0.05, ^##^*p* < 0.01, ^###^*p* < 0.001)

The endogenous respiration of the complexes showed large differences between the YC and the MCI group (*p* < 0.0001), and the OC also had significantly higher endogenous respiration than the MCI group (*p* < 0.0170). There was also a trend between young control and old control (Fig. 4). Within the respiratory chain complexes under maximum substrate supply, no differences were found between MCI and OC, but the values of the MCI group were always higher except for LEAK respiration. Each complex activity was significantly different between YC and the other two groups (Fig. 4).

#### Bioenergetic sex specific differences

The ATP levels of young, healthy subjects exhibited gender-specific disparities, with female subjects demonstrating higher values (*p* < 0.0231) (Table 2). A statistically significant disparity was observed in the respiration of complex IV, with female subjects from the old control group demonstrating higher values compared to their male counterparts (*p* < 0.0266) (Table 3).

**Table 2:** Gender-specific bioenergetic differences in young control. Mean ± SD, *n* = 33; *t*-test with *p < 0.05. *ATP* Adenosine triphosphate, *CS* citrate synthase activity, *CI* complex I, *CII* complex II, *L* LEAK Respiration, *P* coupled respiration, *E* uncoupled respiration

Young control	Female	Male	*p*-value
ATP	0.7820 ± 0.2141*	0.6061 ± 0.1386	0.0231
CS	0.01268 ± 0.0039	0.01237 ± 0.0048	0.8636
Endogenous respiration	2145 ± 562	2401 ± 508.4	0.3179
CI_(L)_	498 ± 256.9	710.3 ± 430.6	0.1304
CI_(P)_	1275 ± 633.1	1423 ± 488.8	0.5997
CI&CII_(P)_	2686 ± 909.3	2410 ± 639.4	0.4916
CI&CII_(E)_	3651 ± 1328	3519 ± 911.8	0.8209
CII_(E)_	1412 ± 495.8	1373 ± 425	0.8619
CIV_(E)_	4451 ± 2028	3445 ± 1034	0.2530

**Table 3:** Gender-specific bioenergetic differences in old control. Mean ± SD, *n* = 33; *t*-test with *p < 0.05. *ATP* Adenosine triphosphate, *CS* citrate synthase activity, *CI* complex I, *CII* complex II, *L* LEAK Respiration, *P* coupled respiration, *E* uncoupled respiration

Old control	Female	Male	*p*-value
ATP	0. 5478 ± 0.1290	0.57 ± 0.1314	0.6640
CS	0.01204 ± 0.0035	0.01349 ± 0.0046	0.3673
Endogenous respiration	2036 ± 543.7	1935 ± 250.7	0.5358
CI_(L)_	334.6 ± 188.4	460.7 ± 243.4	0.1635
CI_(P)_	997 ± 394.9	1052 ± 364.2	0.7217
CI&CII_(P)_	2057 ± 647.9	1943 ± 462.5	0.6135
CI&CII_(E)_	2969 ± 1138	2619 ± 601.4	0.3404
CII_(E)_	1090 ± 401.6	1004 ± 422.5	0.6113
CIV_(E)_	3508 ± 1408*	2398 ± 837.3	0.0266

#### Cognitive sex specific differences

Among participants with MCI, men showed slightly better performance across all four cognitive measures. They had marginally higher scores in IR (*M* = 17.27, *SD* = 4.65 vs. *F* = 17.80, *SD* = 5.70; *p* = 0.78) and DR (*M* = 6.40, *SD* = 2.47 vs. *F* = 5.07, *SD* = 2.02; *p* = 0.12), completed the TMT B faster (*M* = 133.13 s, *SD* = 64.47 vs. *F* = 136.00 s, *SD* = 82.78; *p* = 0.92), and scored slightly higher on Fluency (*M* = 29.80, *SD* = 12.01 vs. *F* = 27.73, *SD* = 11.33; *p* = 0.63). However, none of these gender differences reached statistical significance. In the control group without MCI, the women performed better on most cognitive tests than the men. Only in fluency did men outperform women (M = 41.87, SD = 7.44 vs. F = 39.73, SD = 9.94; p = 0.51). In the other tests, women achieved better results in DR (F = 8.33, SD = 1.40 vs. M = 7.80, SD = 1.37; p = 0.30), TMT B (F = 71.47 s, SD = 17.50 vs. M = 79.53 s, SD = 29.27; *p* = 0.37) and IR (F = 24.73, SD = 2.60 vs. M = 22.60, SD = 3.22; *p* = 0.007). For IR, women performed statistically significantly better than their male counterparts (Table 4).

**Table 4:** Gender-specific bioenergetic differences in MCI. Mean ± SD, *n* = 33; *t*-test with *p < 0.05. *ATP* Adenosine triphosphate, *CS* Citrate synthase activity, *CI* complex I, *CII* complex II, *L* LEAK Respiration, *P* coupled respiration, *E* uncoupled respiration

MCI	Female	Male	*p*-value
ATP	0.4022 ± 0.1231	0.3687 ± 0.0788	0.3830
CS	0.00974 ± 0.002	0.01061 ± 0.0035	0.4843
Endogenous Respiration	1450 ± 624.5	1637 ± 575.5	0.4564
CI_(L)_	115 ± 76.85	242.2 ± 192.5	0.2455
CI_(P)_	887.9 ± 289.5	1136 ± 444.2	0.2063
CI&CII_(P)_	2019 ± 478.7	2355 ± 1030	0.4163
CI&CII_(E)_	2846 ± 547.6	3379 ± 1531	0.3694
CII_(E)_	957.9 ± 506.4	970.4 ± 377.5	0.9563
CIV_(E)_	2811 ± 398.2	3830 ± 1777	0.1290

#### ATP levels and cognitive performance

The association between ATP levels and cognitive performance was analyzed in individuals with MCI and OC. Linear regression analyses with ATP as a predictor for cognitive performance showed a positive association between the ATP levels and Immediate Recall (IR) (β = 0.34, *p* = 0.007). The ATP concentration explained about 11.8% of the variance of the IR (R2 = 0.118, F(1.58) = 7.743; *p* = 0.007). A positive association was also found between the ATP levels and fluency (β = 0.34, *p* = 0.008). The ATP levels explained about 11.4% of the variance in fluency (R2 = 0.114, F(1,58) = 7.495; *p* = 0.008). The results are shown in detail in Table 5.

**Table 5:** Correlation analyses between the ATP levels and cognition (MCI and old Controls): After Bonferroni-Holm method, the significance level is *p < 0.0125. Shown are the standardized regression coefficient (β), the standard deviation (SD), the unstandardized regression coefficient (b), the *t*-value (*t*) and the *p*-value (*p*) for the individual linear regression analyses between the cognitive tests with the ATP levels

Neuropsychological tests	Β	*SD*	*b*	*t*	*p*
IR	0.34	0.123	11.318	2.783	0.007*
DR	0.19	0.129	2.745	1.488	0.142
TMT-B	− 0.16	0.13	− 61.488	− 1.253	0.215
Fluency	0.34	0.124	23.734	2.738	0.008*

**Fig. 5:**
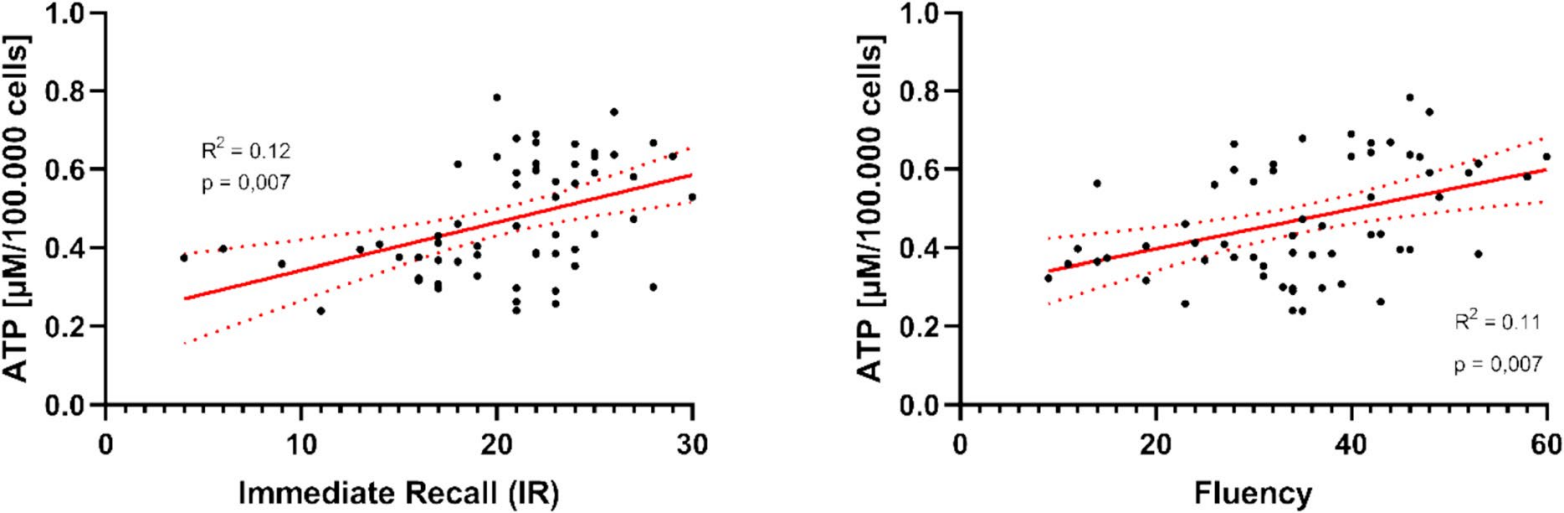
Scatterplots of immediate recall and fluency. Separate linear regression analyses were carried out for each cognitive test and the ATP values of all subjects in the old control group and the MCI-group (*N* = 60). Gender, age, and years of education were controlled for. The slope of the red line represents the strength and direction of the relationship between the cognitive tests and the ATP values

The regression coefficients show a moderate positive correlation with the ATP concentration in both tests (IR and Fluency), which is depicted in Fig. 5. Accordingly, cognitive performance improves with higher ATP levels being present.

For Delayed Recall (R2 = 0.037, F(1.58) = 2.215; *p* = 0.142) and TMT-B (R2 = 0.026, F(1.58) = 1.569; *p* = 0.215), no significant predictive power of the ATP levels was found (Table 5).

#### Endogenous respiration and cognitive performance

The relationship between endogenous respiration and the cognitive tests was also investigated with regard to the cognitive performance of individuals with mild cognitive impairment and older controls. However, no significant correlations were determined (Table 6).

**Table 6:** Correlation analyses between the endogenous respiration and cognition: After Bonferroni-Holm method, the significance level is *p < 0.0125. Shown are the standardized regression coefficient (β), the standard deviation (SD), the unstandardized regression coefficient (b), the *t*-value (*t*) and the *p*-value (*p*) for the individual linear regression analyses between the cognitive tests with endogenous respiration

Neuropsychological tests	Β	*SD*	*b*	*t*	*p*
IR	0.24	0.137	0.002	1.767	0.083
DR	0.15	0.14	0.001	1.052	0.298
TMT-B	0.08	0.141	0.008	0.569	0.572
Fluency	0.13	0.14	0.002	0.894	0.376

## Discussion

Recently, we reported a decrease in ATP levels and impaired mitochondrial respiratory complex activities in freshly prepared PBMC isolated from cognitively healthy elderly individuals compared to young controls [17]. In the present study, we successfully validated age-related mitochondrial dysfunction in frozen PBMC isolated from a new cohort of cognitively healthy elderly and young subjects. Young healthy subjects had higher ATP levels and increased respiratory chain activity compared to healthy old subjects and subjects with MCI. Others also found an impairment of mitochondrial function associated with age. In addition, frailty status was associated with decreased mtDNAcn, imbalance of mitochondrial dynamics, decreased mitochondrial respiration function, and increased ROS levels in PBMC [32]. Mitochondrial dysfunction in PBMC was linked to aging, as shown by decreased mitochondrial ATP production and mitochondrial spare respiratory capacity, as well as an increased ratio of mitochondrial oxidative stress to mitochondrial mass [33]. Gender-specific effects were also observed; healthy women had a higher energy metabolism than men, while no gender-specific effects were observed in subjects with MCI. This observation is consistent with earlier data in which women also had a higher energy metabolism than men in young healthy subjects [34]. Interestingly, healthy women also performed better in the cognitive tests, consistent with their superior bioenergetic profile. Similarly, male subjects with MCI achieved higher cognitive scores, a pattern that was likewise reflected in their bioenergetic measurements. In other studies, older female subjects were also shown to have increased respiration compared to male subjects, with both FAO and glucose-dependent respiration being increased [35].As PBMCs circulate in the blood, they are exposed to various endocrine and paracrine factors, including sex hormones as well as cytokines and chemokines. These may influence mitochondrial function and thus be a potential explanation for the differences [36–38]. Importantly, mitochondrial dysfunction in PBMC was even more pronounced in individuals with Mild Cognitive Impairment. Both endogenous respiration and ATP levels were reduced in individuals with MCI compared to age-matched older controls. Reduced ATP levels in PBMC isolated from the blood of individuals with MCI and age-matched controls were associated with poorer performance on Immediate Recall, which assesses memory function, and Fluency, which assesses verbal fluency and executive function. Interestingly, performance in the Trail Making Test B (TMT-B) was not correlated with ATP levels. The TMT-B engages multiple executive functions, including cognitive flexibility, visual attention, and motor control [21]. These cognitive abilities may be relatively preserved during the early stages of Alzheimer’s disease (AD), in contrast to other executive functions such as those measured by verbal fluency tasks. Notably, both semantic and phonemic fluency have been identified as early and sensitive markers of cognitive decline in AD [39]. The differing cognitive demands of these tests may explain why fluency tasks often detect early impairments more effectively than the TMT-B. Our findings of significant correlations between performance in IR, fluency, and ATP-levels in PBMC of MCI subjects are not consistent with a study by Mahapatra et al. who reported bioenergetic differences in PBMCs between Alzheimer’s patients and healthy controls, but did not report differences between healthy controls and subjects with MCI [40]. Similar to our study, a total of five cognitive tests were performed, including executive function using the Animal Fluency Test (AFT) and episodic memory using immediate and delayed recall tests. The authors report positive correlations between bioenergetic parameters in PBMC, namely mitochondrial maximum and reserve capacity, and cognition. Maximum capacity and reserve capacity in PBMC refer to the ability of cells to produce energy in the form of ATP. An augmented maximum and reserve capacity results in greater ATP production by the cells, particularly in circumstances of stress or elevated energy requirements [40]. Even though the authors could not find any significant differences between NC and MCI, the measurement with the Oxygraph-2 k showed a similar trend as in our measurements, which the authors evaluated in their data as follows. “Together, these results indicate a progressive decline in the activities of specific mitochondrial ETC complexes in PBMCs, from normal control to MCI to dementia.” [40] The fact that we see significant changes between older controls and people with MCI may be due to the severity of cognitive impairment: The mPACC5 test battery used by Mahapatra et al. is excellent for identifying very early, preclinical cognitive changes, whereas the CERAD test we used is more commonly used to diagnose cognitive impairment that has become manifest. Abnormalities in mPACC5 thus indicate a very early stage that may have occurred without changes in CERAD. Abnormalities in CERAD, as we have observed, are usually associated with a more advanced and clinically relevant cognitive impairment. Therefore, one could speculate that in our study the individuals with MCI were more severely affected by cognitive impairment. Moreover, different SUIT protocols were used between the studies, which may also be a reason, although the tendencies of the measurements were the same. Bioenergetic measurements taken with the Seahorse XF Analyzer did not show these differences between NC and MCI.

Although endogenous respiration was reduced in individuals with mild cognitive impairment, it did not show a significant association with cognitive performance. In platelets isolated from blood of AD patients, the mitochondrial respiration rate was significantly reduced [40]. A possible explanation for the observed reduced endogenous respiration could be an impaired glucose metabolism, which has been reported in individuals with MCI and AD [41, 42] which was related to oxidative damage of key proteins involved in glycolysis, the TCA cycle and ATP synthase [43]. Oxidative damage has been found in lymphocytes of both, individuals with AD [44, 45] and with MCI [18]. Our results are in line with studies showing mitochondrial dysfunction in peripheral blood cells of individuals with MCI. Data from mitochondrial respiration parameters reported by Apaiji et al. show that increasing mitochondrial proton leak in PBMC of individuals with MCI was associated with cognitive impairment [18]. However, in this study, ATP levels were not determined. Leuner et al. showed that lymphocytes from individuals with MCI have lower mitochondrial membrane potentials and increased sensitivity to different mitochondrial respiratory chain inhibitors [46]. Sultana et al. showed that mitochondria isolated from lymphocytes of individuals with MCI exhibit increased oxidative stress markers [47]. The stress markers were correlated with cognitive performance, measured with the Mini Mental State Examination. Silva et al. used a cytoplasmic hybrid (cybrid) model created by the repopulation of human teratocarcinoma cells depleted of endogenous mitochondrial DNA with platelets from age-matched controls, MCI, and AD subjects. They found mitochondrial deficits in MCI and AD cybrids as compared with controls, such as a decrease in cytochrome c oxidase activity, a decrease in mitochondrial membrane potential, and in mitochondrial cytochrome *c* content [48].

Since prodromal AD is a common cause of cognitive impairment in MCI, particularly in individuals with amnestic MCI (aMCI), as was the case in the majority of our sample, our data provide evidence for a link between impaired peripheral mitochondrial function and primary AD pathology. Although it is hypothesized that mitochondrial parameters may be suitable as early peripheral markers of the transition between normal aging and dementia [49, 50], the mechanisms by which peripheral mechanisms are related to cognition are still unknown. A recent review discusses whether metabolic disorders mediated by mitochondrial dysfunction, particularly obesity and type 2 diabetes, may be associated with the development of Alzheimer’s disease [49]. Another review discusses the potential role of increased apoptosis, oxidative stress and mitochondrial dysfunction as peripheral markers for detecting AD in lymphocytes [50].

The present study indicates that mitochondrial dysfunction in PBMC is associated with cognitive impairment in old age. PBMC are a valuable source of information about the bioenergetic status of individuals at risk of developing neurodegenerative diseases due to their accessibility. Further studies on larger and more homogeneously stratified samples—for instance, based on amyloid and/or tau-related biomarkers—may provide answers to the question of whether or not measures based on PBMC demonstrate distinct patterns for certain specific pathologies including AD.

## Limitations

Our study has some limitations. One limitation of the study is that there was no neuropsychological assessment of the young control subjects. Since the young control subjects were mainly university students, it was assumed that there would be no cognitive impairment. Even though there was no statistically significant difference in years of education, subjects with MCI had less years of education. However, years of education were included as a covariate in the tests. Another limitation could be the cryopreservation of PBMCs. Cryopreserving and cultivating PBMCs removes them from their original “niche environment”. In addition, it is possible that the concentration of PBMCs in the blood is variable, which can influence the comparability of the results. A FACs analysis of the samples would have been valuable here but could not be carried out due to the limited sample quantity. In addition, PBMCs might have altered glycolytic activity, which is increased in aging immune cells in response to inflammation and nutrition. Lastly, a larger sample size could provide more robust evidence for identifying sex-specific differences.

## Conclusion

Reduced ATP levels in PBMCs isolated from individuals with MCI were found to be associated with cognitive impairment. These findings suggest that PBMCs, due to their accessibility, may serve as informative biomarkers for assessing bioenergetic alterations in individuals at increased risk for neurodegenerative disease.

## Supplementary Information

Below is the link to the electronic supplementary material.

Supplementary file1 (DOCX 266 KB)

## Data Availability

The dataset generated during this study is available from the corresponding author on reasonable request.

## Abbreviations

*AD*: Alzheimer’s disease
*AFT*: Animal Fluency Test
*aMCI*: Amnestic MCI
*ATP*: Adenosine triphosphate
*CERAD*: Consortium to Establish a Registry for Alzheimer’s Disease
*Cr*: Creatine
*CS*: Citrate synthase activity
*DMSO*: Dimethyl sulfoxide
*DPBS*: Dulbecco’s phosphate-buffered saline solution
*DR*: Delayed Recall
*ETS*: Electron transfer system
*FBS*: Fetal bovine serum
*IR*: Immediate Recall
*MCI*: Mild cognitive impairment
*MIR05*: Mitochondrial respiration medium
*naMCI*: Nonamnestic mild cognitive impairment
*OXPHOS*: Oxidative phosphorylation
*PBMC*: Peripheral blood mononuclear cells
*PCr*: Phosphor-creatine
*ROX*: Residual oxygen consumption
*SD*: Standard deviation
*SFT*: Semantic Fluency Test
*TMPD*: Tetramethylphenylenediamine
*TMT-B*: Trail Making Test—Part B
*YC*: Young controls
