# Supplementary material for: Mitochondrial dysfunction in peripheral mononuclear blood cells (PBMC) of individuals with mild cognitive impairment
Source: GeroScience. 2025 Aug 6;48(2):2887–901. doi: 10.1007/s11357-025-01813-4 (PMC12972151; doi:10.1007/s11357-025-01813-4)
Supplement: Supplementary file 1 — Supplementary file1 (DOCX 266 KB) [file 11357_2025_1813_MOESM1_ESM.docx]

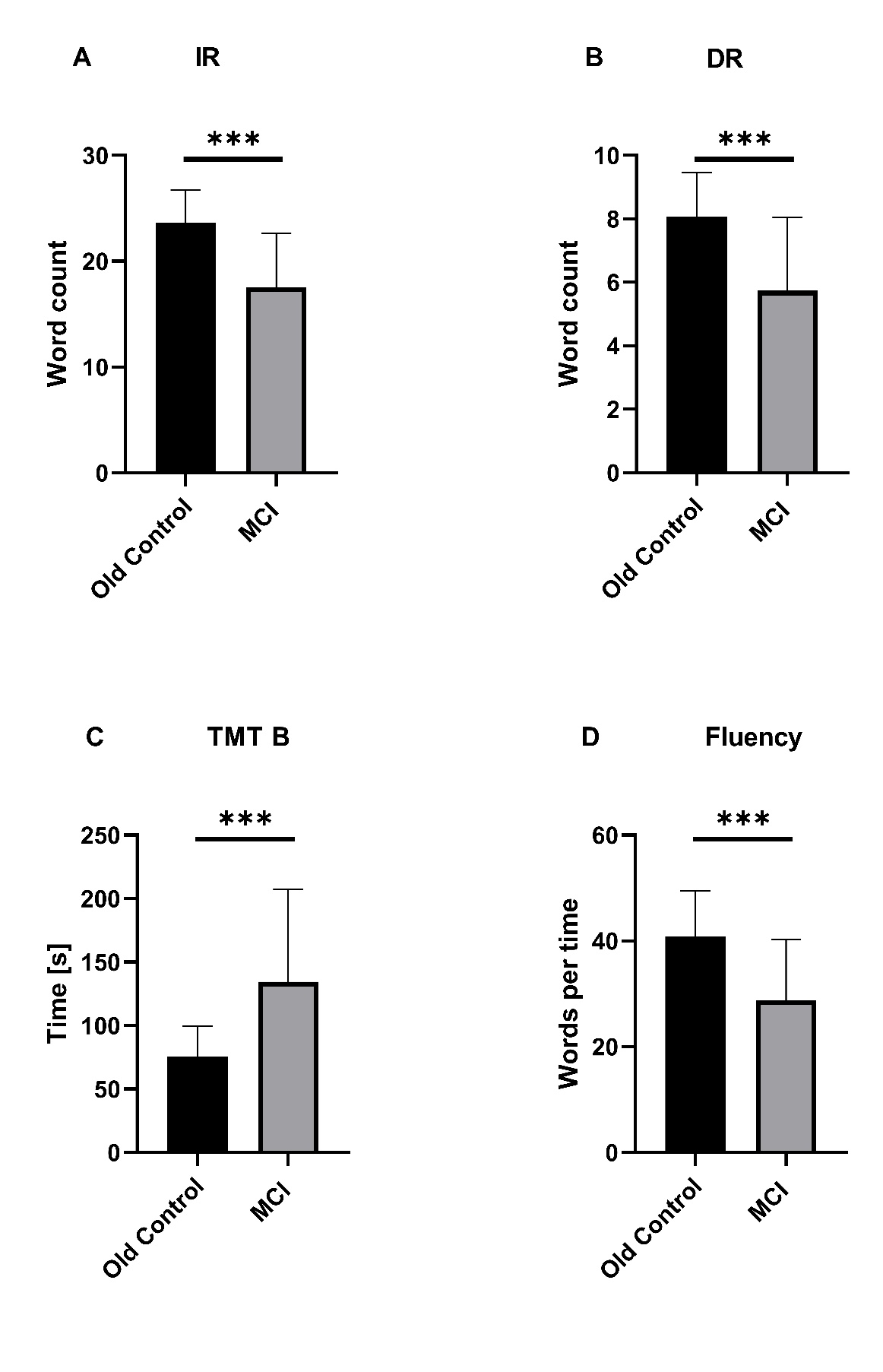


**Supplementary figure 1 psychological tests**

The psychological tests **A** immediate recall **B** delayed recall **C** Trail Making Test - Part B and **D** Fluency were carried out on subjects with MCI (MCI) and their age- and gender-matched healthy controls (Old Control). The sample size for each group was 30 participants, with a gender distribution of 15 males and 15 females. The values denote the mean raw scores (± standard deviation) or the number of subjects. *N* = sample size. The ***p<0.0001 is significantly different compared between MCI and age and gender matched Control Group (unpaired t-test)


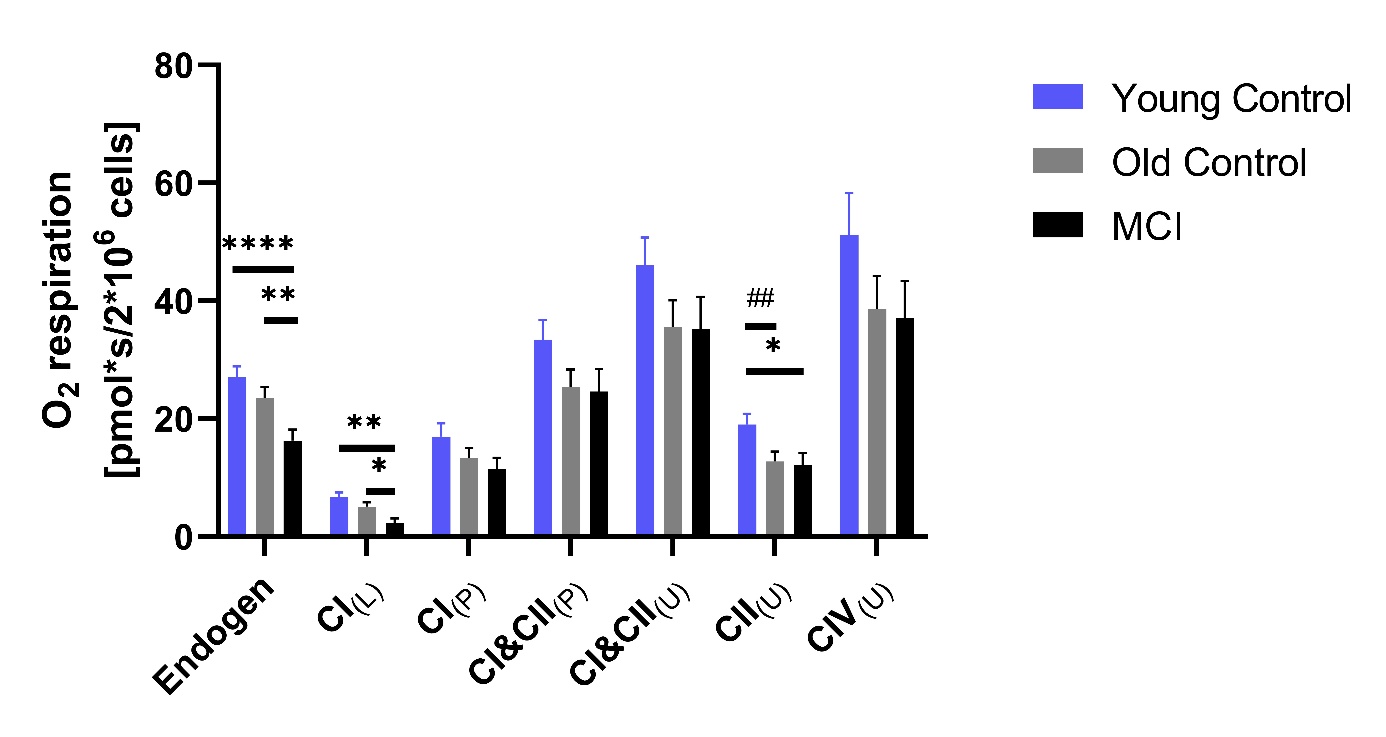


**Supplementary figure 2 Mitochondrial respiration:** PBMC were added to the chambers of the Oxygraph-2k respirometer [4*10^6^cells/ml] to measure the mitochondrial respiratory chain and its complexes. Various inhibitors, substrates and uncouplers were added to differentiate between the individual respiratory chain complexes. Mitochondrial respiration was determined in young control, older controls and people with mild cognitive impairment (MCI). OC = 30, Yc = 30, MCI = 24. The data are given as mean values ± SEM. Significances were determined with an ANOVA (Kruskal-Wallis test or Friedman test). (MCI vs. the control groups: *p < 0.05, **p < 0.01, ***p < 0.001, ****p < 0.0001; Young vs. old control group: ^#^p < 0.05, ^##^p < 0.01, ^###^p < 0.001).
